# Simple plication alleviates physical symptoms in patients with post-gestational rectus diastasis

**DOI:** 10.1007/s10029-023-02814-y

**Published:** 2023-06-24

**Authors:** G. G. Nervil, J. F. Paulsen, J. Kalstrup, S. Deigaard, I. Herbst, S. Lambaa, L. Hölmich

**Affiliations:** 1 Department of Plastic Surgery, Herlev and Gentofte Hospital, Herlev, Denmark; 2 Surgical Department, Herlev and Gentofte Hospital, Herlev, Denmark; 3 Department of Plastic Surgery and Burns Treatment, Rigshospitalet, Copenhagen, Denmark

**Keywords:** Rectus diastasis, Diastasis of the rectus abdominis muscle, Post-gestational diastasis recti, Abdominal wall reconstruction, Plicature

## Abstract

**Purpose:**

To evaluate our surgery for post-gestational rectus abdominis muscle diastasis using slowly absorbable monofilament suture and eight weeks of abdominal binder in terms of recurrence rate, complications, and effect on patients’ physical and cosmetic complaints.

**Method:**

In a retrospective cohort study, all 44 patients operated between 2014 and 2020 were invited to a follow-up using ultrasound, clinical examination, and questionnaires regarding symptoms before and after surgery.

**Results:**

89% of invited patients participated, with a median follow-up of 36 months. There was one recurrence caused by severe postoperative nausea and vomiting, which was the most common complication. Most procedures were safe, but two patients experienced Clavien–Dindo grade 3 complications. Patients reported feeling limited or taking precautions after surgery for a median of 8.5 months. Of all included patients, four responded that the operation did not alleviate their primary complaint. The remaining 35 patients (90%) experienced complete or partial alleviation of their primary complaints and would undergo the procedure again if needed.

**Conclusion:**

Post-gestational diastasis recti can be associated with a large number of physical symptoms and functional complaints and can safely be operated using a single running plication of the anterior rectus fascia with a slowly absorbable suture, with fair cosmetic results, excellent effect on symptoms, few complications and high levels of patient satisfaction. Future research must determine which symptoms and findings should indicate surgery.

**Supplementary Information:**

The online version contains supplementary material available at 10.1007/s10029-023-02814-y.

## Background

Diastasis of the rectus abdominis muscles (rectus diastasis, RD) is a physiological part of pregnancy and the immediate post-pregnancy period, caused by a hormonal thinning and relaxation of the linea alba and abdominal musculature to allow for the growing fetus [1]. The exact definition of diastasis between the rectus abdominis muscles is still debated [2–6] but has been found to be present in 100% of third-trimester pregnant women [1]. The condition regresses after delivery, but in a third of women, the condition has been described to persist after a year [7]. A recent guideline from the European Hernia Society recommends defining rectus diastasis (RD) as a widening of the linea alba exceeding 2 cm. It suggests a classification system that includes type, inter-rectus distance, and presence of concomitant hernia [8]. The guideline has primarily weak recommendations for pre- and post-surgical management and outcome measures due to the limited literature and research in this area [8]. RD has generally been regarded as a condition women must accept when choosing to give birth. With the limited research in the field, the consequences of having diastasis of the abdominal muscles are unclear and are still being discussed [9–12]. Besides a protrusion of the abdomen, symptoms of reduced muscle function, pain and vulnerability have also been described, along with reduced core muscle functions with secondary pain in the back and pelvic floor dysfunction [12–20]. The proposed associated symptoms could potentially be alleviated with non-surgical treatment (physiotherapy, specialized training protocols, abdominal binder, etc.), but there are mainly small studies and diverging recommendations about the possible treatment options [18, 21–24]. The most controversial issue regarding physiotherapy is whether the rectus abdominis muscles should be strained or not during exercise [18, 23–25]. Despite the lack of scientific evidence, there is increasing activity on social media with recommendations and exercise guidelines for RD by physiotherapists, but also in a growing number of unauthorized “experts”. Only a few studies have tested training vs. no training and found no certain effect on the symptoms associated with RD [26] or on recovery after birth and width of the diastasis [18]. Recently, Danish women have been given the option of tax-paid subsidy for physiotherapy for symptoms associated with rectus diastasis.

Where the evidence for non-surgical treatment is very sparse, outcomes and complications after surgical treatment has, within recent years, been a topic of scientific research [27, 28]. RD can be corrected surgically [29, 30], which has been performed for aesthetic purposes in combination with abdominoplasty within plastic surgery for many years [31, 32]. Our department has seen an increasing number of patients seeking surgery due to functional problems, mainly with abdominal and/or back pain and lack of core stability.

A literature review concludes that surgery can alleviate physical symptoms but also addresses the lack of valid instruments for assessing indications and outcomes [33], and the need for research measuring patient reported outcomes is advocated in another recent review [34].

The aim of this study was to evaluate the results of our practice within the last seven years, where women with clinical RD and functional complaints considered associated with RD have been operated with abdominoplasty and simple plication. The study will report the recurrence rate and complications, the effect on physical and cosmetic complaints, and patient satisfaction.

## Methods

All 44 patients operated for post-pregnancy diastasis recti with simple plicature and no use of mesh at our institution from 2013 to May 2020 with a minimum of six months of follow-up, who could read Danish, were invited to participate in this retrospective cohort study. An invitation was sent by secure digital mail and followed up with a subsequent phone call to schedule their appointment. Participation included a questionnaire and a physical examination with ultrasound examination of the linea alba, done by one of two independent investigators, and clinical photos by a photographer. The linea alba area was examined in its entire length and the width 3 cm cranial to the umbilicus, at the umbilicus and 2 cm caudal to the umbilicus was measured in cm with one decimal, along with the widest measurement if this was different from any of these three standardized locations. The questionnaire was study specific and exploratory and had items concerning recalled symptoms before surgery, current symptoms, and the patient’s satisfaction with the physical and cosmetic result of the surgery. In addition to the study specific questionnaire the Swedish Ventral Hernia Pain Questionnaire (VHPQ) was used, as it has previously been tested in a population of RD patients and found to be valid in preoperative evaluation for deciding surgery [35]. Before using the translated VHPQ, it had undergone a linguistic validation process with the Swedish author group's acceptance of the translated questionnaire.

Information about the pre-operative diastasis, operation, and postoperative complications was obtained from the patient records after participant enrolment. The patients were classified according to the European Hernia Society classification [8]. Complications were categorized according to the Clavien–Dindo classification [36].

Study data were managed using the REDCap database (Research Electronic Data Capture, REDCap Consortium) hosted by The Capital Region of Denmark.

The primary study outcome was recurrence of RD, defined as a post-operative inter-rectus muscle diastasis of more than 3 cm. Secondary outcomes included postoperative width of the linea alba, rate of complications, effect on physical and cosmetic complaints, and patient satisfaction.

### The patient population

Patients referred to our department with symptomatic RD were seen for pre-operative evaluation by one of the two consultants. Patients with relevant physical complaints, a palpable diastasis of 3 cm or more, and a history of relevant training were candidates for surgery. The abdominal wall function was examined in an upright and supine position, asking the patient to demonstrate withdrawal of the abdomen in the upright position, and lifting the head from the couch in the supine position. Clinical measurements of RD were performed during the examination using either finger-span measure or ruler. If the patient had a significant lack of muscle control, she was generally referred to physiotherapy for at least three months, and the situation was re-evaluated before offering surgery. In addition, the patient had to be at least one year post-pregnancy, without the intention of further pregnancies, have a normal BMI, and be a non-smoker. In the case of concurrent hernia, a higher BMI was accepted, and the abdominal wall was examined pre-operatively with ultrasonography and/or a CT scan by radiologists. The linea alba area was described, including width measurements, generally at the same levels as in the clinical examination.

### Surgical technique

The surgery was performed under general anesthesia by the same two consultants performing an abdominoplasty with a simple plicature of the anterior rectus sheet using a standard procedure. The horizontal incision was placed as low as possible (just above the symphysis pubis), taking skin excess into consideration. In case of little or no skin excess, the horizontal incision was placed higher than usual, or an inverted T-scar was used. The abdominal skin and subcutaneous tissue was dissected from the muscle fascia circumcising the umbilicus. In the upper midline, the dissection was carried on toward the ribs and the xiphoid process until obtaining a minimum margin of 2–3 cm lateral to the diastasis while preserving the perforating vessels to the skin along the lower ribs. In the lower abdomen, the dissection was continued out more laterally and in a slightly more superficial plane to spare lymphatics and reduce seroma formation.

The rectus diastasis was closed with a simple running plication of the anterior rectus abdominis muscle fascia using a slowly absorbable monofilament loop suture (PDS^®^ 0). Relaxation of the patient was used during the plication by one of the surgeons. Plication was done from the xiphoid to the symphysis regardless of the extension of the diastasis with stitches about 1 cm long and with 1 cm in between (large bites); one surgeon placed the stitches vertically and the other horizontally. At the level of the umbilicus, the running suture was either discontinued (during the first part of the period) or continued passing the umbilicus below the facia along either side of this. Any midline hernias were attended as necessary. Small hernias were attended with dissection and simple reposition of the content (predominantly just pre-peritoneal fat), closure of the defect in the fascia with either Vicryl^®^ or PDS^®^, followed by the plication which served as further support for the hernia. In case of more or larger hernias and if a hernia was present outside the linea alba area, one of two abdominal surgeons repaired the hernia with a mesh in addition to the plicature. Patients operated using mesh were excluded for this study.

The excess skin was excised, a new entry for the umbilicus was created, and the incisions were closed in 2–3 layers. A suction drain was placed along with (since 2018) a subcutaneous pain catheter for local anesthetics (ropivacaine) for up to three days postoperatively. The patient was dressed in an abdominal binder at the end of the procedure. A urinary catheter was used until the evening or the next morning, depending on how well the patient was mobilized.

### Post-operative regime

The patients were hospitalized as long as pain was a significant problem and until they were mobilized and able to manage on their own, usually 2–3 days. The patient was instructed to wear the abdominal binder day and night for four weeks and during the day for an additional four weeks. They were instructed not to strain the abdominal muscles for eight weeks and avoid lifting more than a few kilos—including not lifting their children. Skin sutures around the umbilicus were removed two weeks postoperatively by nurse practitioners. The first planned follow-up with the surgeon was two months after surgery, where physiotherapy-guided exercises were initiated with slowly increasing load on the abdominal muscles. Final check-up was done six months after surgery, at which point the patient was allowed free activities, including strenuous training.

### Statistics

All descriptive statistical analysis was done using IBM SPSS Statistics 25.0 (IBM Corporation, Armonk, NY). Questionnaire data regarding pre- and postoperative evaluations was compared using *X*^2^ or Fischer’s exact test with a statistical significance level of 0.05.

The pre-operative measurements of the widest distance of the RD were obtained from patient records using the best available sources, defined as CT scan followed by ultrasonography, peri-operative measurement, and clinical pre-operative measurement in that order. The average value was chosen for analysis if this measurement was indicated as a range.

As this study is explorative in nature, the questionnaire included several free-text fields where the participants could answer by writing in free form. These answers, including the patient’s primary complaint or reason for wanting surgery, were subsequently categorized by the study group for statistical analysis.

## Results

Of 44 eligible patients, 37 participated with both questionnaire and physical examination. Of the seven non-participating patients, four did not respond to the invitation, one was excluded due to language barrier, and two participated only with response to the questionnaire but were not seen for physical examination. The participation rate was 89% by questionnaire and 84% by physical examination.

The patients reported a median of two pregnancies (range 2–5) before their surgery for RD and an average baby weight of 3850 g (range 2300–5004 g) per singleton pregnancy. Eight women (21%) had had gemelli pregnancies with an average combined baby weight of 5124 g (range 4400–6200 g). The patients most often reported that symptoms arose after their second pregnancy (50%). Twenty-two patients (56%) had had at least one cesarean section and the median number of caesarean deliveries was 1. All patient demographics are presented in Table 1.

**Table 1:** Participant Demographics

Number of respondents	39
Demographics	
Age at the time of surgery, years, mean (range)	37.1 (26–49)
BMI before surgery, mean (range)	21.9 (18.8–28.1)^a^
Pregnancy history	
Number of pregnancies, per woman, median (range)	2 (2–5)
1	0
2	25 (64%)
3	11 (28%)
4	2 (5%)
5	1 (3%)
Singleton and twin of pregnancies	
Singleton pregnancies	88
Gemelli pregnancies	8
Weight of baby	
Singletons, g, mean (range)	3849.9 (2300–5004)
Gemelli babies combined, g, mean (range)	5124.4 (4400–6200)
Number of pregnancies before symptoms, median (range)	2 (1–3)
Number of cesarean sections, median (range)	1 (0–4)
Number of women having had at least one cesarean section	22 (56%)
Concurrent hernia	12 (31%)
Umbilical hernia	10 (26%)
Other linea alba hernia	6 (15%)
Other abdominal wall hernia (incisional, etc.)	0
Largest hernia diameter, mm, mean (range)^b^	11.0 (5–25)
EHS RD classification^c^	
T1D1H0	0
T1D1H1	0
T1D2H0	9
T1D2H1	4
T1D3H0	18
T1D3H1	8
Time to surgery	
Time from start of symptoms till referral to plastic surgery, years, median (range)	4.0 (0–21)
Follow-up time, months, median (range)	35.9 (5.2–79.8)

*T1* RD after pregnancy, *D1* Inter-rectus diastasis > 2–3 cm, *D2* Inter-rectus diastasis > 3–5 cm, *D3* Inter-rectus diastasis > 5 cm, *H0* Without hernia, *H1* Hernia present

^a^Data available for 35 patients

^b^Data available for 10 of the 12 concurrent hernias

^c^European Hernia Society guidelines for classification of rectus diastasis

The widest distance between the rectus abdominis muscles pre-operatively was on average 5.6 cm (range 3.4–12.5 cm), based on the best available measurement (Table 2). At follow-up, the mean distance between the medial rectus borders was 1.7 cm (0.1–3.6 cm).

**Table 2:** Pre- and postoperative rectus diastasis measurements and location of broadest diastasis

Location of measurement	Inter-rectus distance before plication, *n* = 39			Inter-rectus distance after plication, *n* = 37
	Clinical, cm (range)	Peri-Operative, cm (range)	Imaging cm (range)	Ultrasound^d^ cm (range)
3 cm Supra-umbilically	–	–	–	1.0 cm (0.1–2.3)
At the level of the umbilicus	–	–	5.3 cm (3.5–9.3)^a^	1.5 cm (0.2–3.6)
2 cm Infra-umbilically	–	–	–	1.0 cm (0.1–2.7)
At the broadest location	5.5 cm (2.0–10.0)	6.5 cm (4.0–12.0)^c^	5.5 cm (3.4–11.2)^b^	1.7 cm (0.7–3.6)^e^
At the broadest location, using the best measurement available	5.6 cm (3.4–12.5)			1.7 cm^e^ (0.7–3.6)

Location of the broadest inter-rectus distance	*n* (%)	*n* (%)
Supra-umbilical	9 (31)	9 (24)
Peri-umbilical	21 (54)	26 (70)
Infra-umbilical	1 (3)	2 (5)
Unknown (data not available)	8 (21)	

All measurements reported are the median measured in centimeters

Data too scarce or unsystematic to report

^a^Data available for 21 patients

^b^Data available for 22 patients

^c^Data available for 31 patients

^d^Data available for 37 patients

^e^1.7 cm (0.7–2.7) if excluding the patient with immediate post-surgical recurrence

The median follow-up time was 36 months (range 6–80 months) for the 37 re-examined patients, of whom one had a clinical and subjective recurrence of the diastasis. This patient suffered severe postoperative nausea and vomiting and felt something in the upper abdomen rupturing during vomiting, corresponding to a finding of recurrent diastasis in this area shortly after the procedure. Her initial diastasis was 8.8 cm with a concurrent hernia (EHS RD class T1D3H1). At the study follow-up, the RD measured with ultrasonography was 3.6 cm in her upper abdomen, and the patient was awaiting re-operation. The postoperative mean diastasis width can be seen in Table 2.

Complications to the procedure were generally minor and the most common complication was post-surgical nausea and/or vomiting. Two patients account for all the Clavien–Dindo (CD) grade 3 complications (Table 3). One patient was unaware of a hematological condition that made her susceptible to bleeding, and immediate re-operation for hematoma (CD grade 3B) and blood transfusions were necessary. Another patient developed a small hematoma diagnosed six weeks post-surgery, which was operated in general anesthesia (CD grade 3B), developed a subsequent infection and was administered intravenous antibiotics (CD grade 2).

**Table 3:** Surgical information and postoperative complication rates (*n* = 39)

Surgical information	*n* (%)
Incision type	
Horizontal	18 (46)
Horizontal and vertical (anchor)	21 (54)
Number of drains	
0	8 (20)
1	25 (64)
2	7 (18)
Time to discharge, days, median (range)	3 (1–6)

Complications	Clavien–Dindo grade	During hospitalization *n* (%)	After discharge *n* (%)
Recurrence of diastasis^a^		1 (3)	
Recurrence of hernia			0
Post-operative pain		6 (15)	3 (8)
	1	5 (13)	2 (5)
	2	1 (3)	1 (3)
Bleeding or hematoma		2 (5)	1 (3)
	1	1 (3)	
	3B^b^	1 (3)	1 (3)
Infection			6 (15)
	1		1 (3)
	2		4 (10)
	3A^c^		1 (3)
Seroma		1 (3)	1 (3)
	1	1 (3)	
	2		1 (3)
Nausea and/or vomiting		13 (33)	
	1	13 (33)	
Wound healing complications			5 (13)
	1	1 (3)	5 (13)
Ventilator-associated pneumonia (VAP)	2	1 (3)	1 (3)
Other complications^d^		6 (15)	
	1	4 (10)	
	2	2 (5)	
Unsightly scarring^a^^5^			9 (24)
Additional scar corrections necessary			13 (31)
Done before the time of the follow-up examination			8 (19)
Patients still waiting at the time of the follow-up examination			5 (13)
The period where the patient felt limited because of surgery, months, median (range)			8.5 (2–48)^f^

^a^Data available for 37 patients

^b^Treatment of one patient with an unknown bleeding disorder (immediate reoperation and blood transfusions, tranexamic acid and IV fluids) and one patient treated with operative extirpation of a hematoma

^c^One patient treated with drainage of the infected cavity

^d^Coughing, reddening of the thigh, headache, constipation, flushing of face and chest

^e^Includes broadening of the scar, hyperpigmentation or hypertrophic scar tissue

^f^13 patients still took precautions at the date of follow-up, data available for 38 patients

Postoperative pain was completely gone in 38% of the patients three months after surgery and for an additional 31% within six months. Five patients (13%) reported that their postoperative pain lasted for a year, and one reported pain lasting two years (VHQP questionnaire answers available as supplementary material in Table 5). Patients reported feeling limited or taking precautions for a median of 8.5 months after their surgery.

Asked about their primary reason for seeking surgery (and only allowing for one reason), 56% reported pain as the main reason. Of these, 13 patients reported pain in the back or the lower back, five reported pain in the abdomen or the abdominal wall, and four reported pain elsewhere or did not define their pain. The remaining primary reasons for seeking surgery were categorized as cosmetic issues (18%), discomfort (15%), and other reasons (10%) (Table 4). The participants’ evaluation of the effect of surgery on their primary complaint was evenly distributed across the categories of the EHS RD classification, with most patients reporting complete or partial alleviation of their primary complaint. However, four patients (10%) reported no effect on their primary complaint. No obvious explanation for this could be found in the data, with two of these patients having a diastasis of EHS class T1D2H0 and the two others having a diastasis of EHS class T1D3H0 and T1D3H1, respectively.

**Table 4:** Study-specific questionnaire responses (*n* = 39)

	Before surgery *n* (%)	After surgery *n* (%)
Physical symptoms		
Pain in the abdomen	23 (59)	8 (21)*
Hernia-like pain or discomfort	30 (77)	4 (10)*
Feeling unprotected or weak spot in the stomach wall^a^	20 (51)	7 (18)*
No response	17 (44)	0
Difficulty activating stomach muscles	35 (90)	9 (23)*
Difficulty withdrawing the navel	31 (79)	3 (8)**
Difficulty getting up from prone	27 (69)	5 (13)*
Discomfort during meals	13 (33)	5 (13)
Discomfort when touched around the navel or diastasis^b^	26 (67)	21 (54)
No response	0	2 (5)
Difficulty increasing stomach pressure, as when on the toilet^b^	10 (26)	2 (5)*
No response	2 (5)	1 (3)
Discomfort or pain when having sexual intercourse^b^	9 (23)	2 (5)
No response	1 (3)	2 (5)
Back pain	30 (77)	8 (21)*
Lower back pain	32 (82)	17 (44)*
Pelvic pain	20 (51)	7 (18)*
Tiredness in the back muscles	36 (92)	12 (31)**
Other functional or physical symptoms	12 (31)	1 (3)^2^*
Cosmetic complaints		
Dissatisfied with looks of the abdomen	39 (100)	15 (38)**
Protruding abdomen	37 (95)	9 (23)**
Looking pregnant	36 (92)	3 (8)**
Extra skin on the abdomen	28 (72)	6 (15)*
Difficulty hiding the abdomen in clothing	38 (97)	6 (15)**
Uncomfortable to be seen without clothing	37 (95)	15 (38)**
Has received comments about the abdomen	33 (85)	4 (10)**
Other cosmetic complaints	12 (31)	2 (5)*
Limited in activities		
Work activities	15 (38)	4 (10)*
Spare time activities	24 (62)	4 (10)**
Training/exercise	29 (74)	9 (23)*
Use of medication because of your complaints	13 (33)	3 (8)*
Analgesics	10 (26)	2 (5)*
Laxatives	6 (15)	2 (5)
Other^c^	2 (5)	1 (2)
The most important symptom/reason for wanting surgery, and patient-reported effect of surgery on that symptom/reason		
Pain	22 (56)	
Complete alleviation		11 (28)
Partial alleviation		9 (23)
No effect		2 (5)
Cosmetic	7 (18)	
Complete alleviation		4 (10)
Partial alleviation		2 (5)
No effect		1 (3)
Discomfort or lack of core muscle control	6 (15)	
Complete alleviation		2 (5)
Partial alleviation		3 (8)
No effect		1 (3)
Other	4 (10)	
Complete alleviation		1 (3)
Partial alleviation		2 (5)
No effect		0
No answer		1 (3)

Patient reported effect of surgery	
Effect of surgery on symptoms	
Very good	19 (49)
Good	17 (44)
Less good	2 (5)
Hardly any or no effect	0
Don’t know	1 (3)
Effect of surgery on the shape of the abdomen	
Very good	21 (54)
Good	13 (33)
Less good	2 (5)
Hardly any or bad	3 (8)
Don’t know	0
Sensitivity around the navel	
Normal	3 (8)
Decreased	19 (44)
Discomfort when touched	11 (28)
No sense of touch	6 (15)
Satisfaction with the scarring	
Very satisfied	13 (33)
Satisfied	18 (46)
Less satisfied	4 (10)
Unsatisfied	4 (10)
Don’t know	0
Patients who would undergo the surgery again in case of recurrent diastasis and symptoms	35 (90)
Patients who would recommend the surgery to others	35 (90)

^a^The question about pre-operative feeling of unprotectedness or weak spot was initially forgotten and distributed later

^b^Answers not mandatory

^c^Before surgery: Anxiolytics (diazepam), muscle relaxant (chlorzoxazone) and pantoprazole

After surgery: Pantoprazole; * and ** indicates statistically significance at a level of *p* < 0.05 and *p* < 0.001 on *X*^2^ or Fisher’s Exact test

Of the 39 patients that responded to the questionnaire, 38 patients (92%) reported the surgery as having a good or very good effect on their symptoms, 37 patients (88%) had complete (46%) or partial (41%) alleviation of their primary complaint, and 31 patients (79%) were satisfied or very satisfied with the cosmetic result. In total, 35 (90%) would undergo the surgery again and recommend it to others. See Table 4 for more patient-related outcomes.

## Discussion

In this study of the outcome of surgery for symptomatic post-gestational rectus diastasis with abdominoplasty and simple plication of the anterior rectus sheet, we found few complications to the procedure. The vast majority of patients reported a good effect on their primary complaint. We found a significant reduction in the prevalence of almost all reported symptoms after surgery, and most of the patients would undergo the procedure again if needed. There was one patient with recurrence of the diastasis.

Our results show a similar or lower rate of recurrence than other studies using a similar type of suture repair [37–39] and a lower rate of seroma [10]. We believe the good results, including the low rate of seroma, are likely to be due to a combination of elements: The surgical technique, the use of an absorbable suture, no use of mesh, the use of suction drainage, the use of abdominal binder, and the strict post-surgical regime. Both the use of drains and compression bandage is debatable [40, 41].

In our cohort, the most common complication was nausea and/or vomiting, occurring in 33% of the patients. In one patient with a clinical recurrence of the diastasis, postoperative vomiting caused the recurrence. Reduction of post-operative nausea and/or vomiting is important, both for patient comfort and morbidity, and standard use of a pain catheter with ropivacaine seems to have reduced use of oral morphine and nausea (results not reported here). As many as 93% of the patients had either decreased (44%), disturbed (28%), or complete loss of sensation (15%) around the umbilicus following the procedure, despite the long median follow-up time. This complication is not usually reported but appears to be very common after this procedure. Furthermore, dissatisfaction with the scars was not rare (20%), and many had to have their surgical scars revised (dog ear corrections). Two patients had Clavien–Dindo grade 3 complications, which is higher than others have reported [10].

The prevalence of almost all symptoms experienced to be associated with RD by the participants were reduced significantly by the surgery. Our findings are in line with the few other similar studies in this area. RD can indeed be a disabling condition and is not merely a cosmetic issue [15, 42], despite it being classified as such in the current Common Procedural Terminology coding [43]. Almost all reported symptoms could be related to the lowered ability to provide a firm abdominal wall with intact trunk stability. It is easily understandable that thinning and distention of the linea alba to a degree where bowel movement can easily be seen under the skin is able to produce symptoms similar to hernias, though without the risk of incarceration. It is likewise understandable that malfunction of the abdominal wall must influence other muscles engaged in the core stability, including the muscles of the back. Some authors have also focused on the pelvic muscle function, including urinary incontinence, which some have found to be affected by RD [35], though others have found no such association [44]. We did not investigate these particular symptoms.

The patients reported taking precautions or feeling limited in their daily activity for a median of 8.5 months after the procedure. This is a significant finding. Not only is this information crucial for clinicians to include when informing their patients before surgery, as the procedure will impact their daily activity much longer than most would expect. But the finding is also essential for future studies, as a more than six months may be needed for follow-up. In our population, 77% of patients report being pain-free and not feeling limited 12 months post-surgery.

We were surprised to learn that 18% of our patients reported that the cosmetic issues with RD were their primary driver for seeking treatment. We intended to only operate patients with physical problems, as cosmetic surgery is not offered in our health service system. This underlines that better methods for patient selection are needed. Although no formal criteria of a minimum diastasis width were established in our department, no patients with a diastasis of less than 3 cm were operated. This is in concordance with the newly published European and Swedish guidelines for the management of RD [8, 22], although the Swedish guideline generally only recommends surgery if the diastasis is 5 cm or above, except in case of severe bulging where a 3 cm limit may be used, or in case of concomitant hernia where no specific limit for RD is needed [22]. A minority of our patient population had concomitant small midline/umbilical hernias, which were remedied with suturing alone as the plicature served as reinforcement, eventually leading to a solid fibrosis with several layers of the linea alba folded over itself. We found no hernia recurrences in our population. Our sample size is too small and suffers from selection bias to be used for the analysis of any correlation between risk factors such as concomitant hernia-repair, pregnancy history, way of birth, and other demographic variables.

No substantial evidence exists for physiotherapeutic treatment as a cure for RD. Increasingly, patients seek private sector training programs, but there is currently little evidence for or against the efficacy of activating only the transversus abdominis muscle [18, 45]. Such programs place physical restrictions on daily activities, exercise, and quality of life. A Norwegian study showed no effect of physiotherapist-guided training on physical symptoms or objective findings [26], while an Egyptian study found a significant effect on rectus diastasis and quality of life by adding specific core training and an abdominal binder to regular abdominal exercises [46]. A Swedish randomized trial found no differences between two surgery groups, and all participants in the training group opted for surgery due to unsatisfactory results [15].

Our study is limited by the number of included patients and by not having recorded the information prospectively. Among strengths are that the pre-operative evaluation and the surgical procedures were standardized and done by only two surgeons in the department and that the postoperative regime was strict and standardized. The retrieval of information from the medical records and the postoperative evaluation was done by independent investigators, including clinical follow-up and diastasis measurement using ultrasound at standardized locations. Additionally, the cohort has a long median follow-up time, demonstrating that a one-layer plicature with an absorbable suture is indeed feasible and that good long-term results can be obtained.

The prevalence of symptomatic post-gestational RD among women is unknown. A specific width of RD is practical in establishing guidelines. Still, it may not be representative of the extent of the symptoms associated with RD, as a recent more extensive study of 266 women has found no correlation between the width of the diastasis and back pain or functional disability [47]. A classification proposal was published in 2019 based on the available literature, focusing on the structures in the front of the abdominal wall [48], but perhaps the connective tissue strength and thickness, or the relative width of the RD compared to the abdominal circumference, could also be important measures. More research into the matter is needed. We need to establish how common post-gestational RD is and what symptoms can be ascribed to the condition, including how often the condition is asymptomatic. We need better tools for the evaluation of the abdominal wall function, and better evidence is required to determine if a patient will benefit from surgery.

## Conclusion

This study demonstrates that post-gestational RD can be associated with a large number of physical symptoms and functional complaints. RD can safely be operated using a running plication of the anterior rectus with a slowly absorbable monofilament suture when adhering to a strict postoperative regime with limited physical activity during healing. Adherence to our protocol provided fair cosmetic results, excellent effect on symptoms, few complications, and high levels of patient satisfaction.

**Fig. 1:**
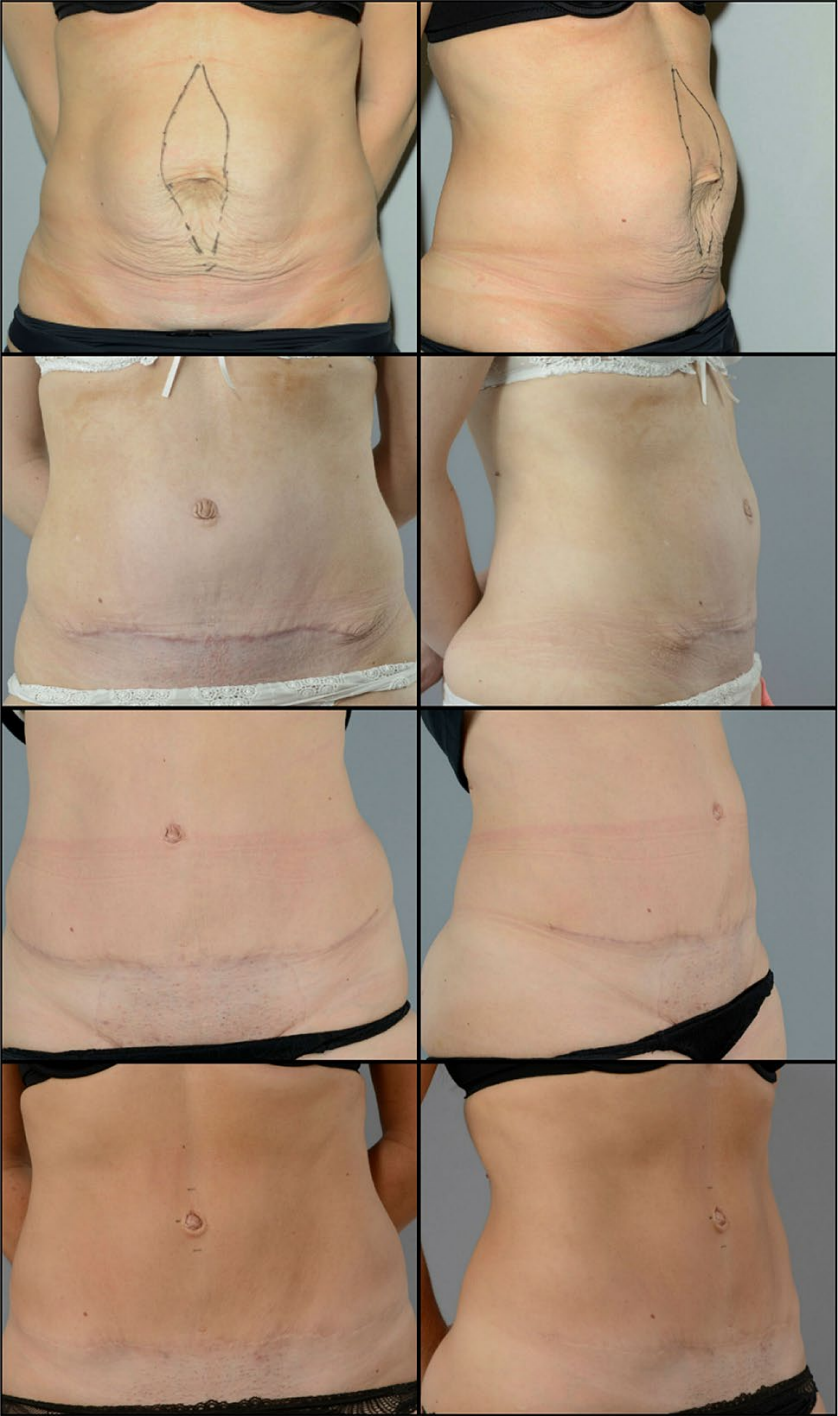
Clinical Images pre- and post-surgery. The patient is a woman in her late thirties with a 6 cm diastasis after four pregnancies. Symptoms including abdominal pain when eating, back pain due to constantly keeping tension in her abdominal muscles and receiving comments from others about expecting a fifth child. Diastasis plicated with PDS 0 loop suture. Excess skin protrusions at the end of the scar removed 9 months after primary surgery. Pictures (top to bottom row) showing her abdomen pre-operatively, 2 months post-surgery, 1 year and 5 years post-surgery

## Supplementary Information

Below is the link to the electronic supplementary material.

Supplementary file1 (DOCX 28 KB)

## Data Availability

According to Danish data regulations, we are not allowed to share the data.
