# Supplementary material for: Simple plication alleviates physical symptoms in patients with post-gestational rectus diastasis
Source: Hernia. 2023 Jun 24;27(4):957–68. doi: 10.1007/s10029-023-02814-y (PMC10374809; doi:10.1007/s10029-023-02814-y)
Supplement: Supplementary file 1 — Supplementary file1 (DOCX 28 KB) [file 10029_2023_2814_MOESM1_ESM.docx]

**Additional Online Materials**

**Table 5.** Ventral Hernia Pain Questionnaire (n=39)

|  | n (%) |
| --- | --- |
| **2. Describe the pain you experienced from your hernia prior to surgery** |  |
| A, No pain | 5 (13) |
| B, Pain that could be easily ignored | 8 (21) |
| C, Pain that could not be ignored but did not influence daily activities | 18 (46) |
| D, Pain that inhibited most daily activities | 6 (15) |
| E, Pain that required rest or bed rest | 2 (5) |
| F, Pain so severe that you were forced to seek medical attention | 0 |
| **3. Describe the abdominal pain you experience right now following surgery** |  |
| A, No pain | 28 (72) |
| B, Pain that could be easily ignored | 5 (13) |
| C, Pain that could not be ignored but did not influence daily activities | 2 (5) |
| D, Pain that cannot be ignored, which affects concentration and daily activities | 3 (8) |
| E, Pain that inhibited most daily activities | 0 |
| F, Pain that required rest or bed rest | 1 (3) |
| G, Pain so severe that you were forced to seek medical attention | 0 |
| **4. Describe your abdominal pain when most intense during the last week** |  |
| A, No pain | 21 (54) |
| B, Pain that could be easily ignored | 4 (10) |
| C, Pain that could not be ignored but did not influence daily activities | 7 (18) |
| D, Pain that cannot be ignored, which affects concentration and daily activities | 3 (8) |
| E, Pain that inhibited most daily activities | 2 (5) |
| F, Pain that required rest or bed rest | 1 (3) |
| G, Pain so severe that you were forced to seek medical attention | 1 (3) |
| **5. If you no longer have pain in the operated area, try to recall when your abdominal pain stopped. After answering this question, go skip to question 16** |  |
| A, I still have abdominal pain | 4 (10) |
| B, Pain in the operated area stopped within 1 month following surgery | 4 (10) |
| C, Pain in the operated area stopped within 3 month following surgery | 11 (28) |
| D, Pain in the operated area stopped within 6 month following surgery | 12 (31) |
| E, Pain in the operated area stopped within 1 year following surgery | 5 (13) |
| F, Pain in the operated area stopped within 2 years following surgery | 1 (3) |
| G, Pain in the operated area stopped recently | 2 (5) |
| **6. How often have you felt abdominal pain in the operated area in the last week?^1^** |  |
| A, A few times during the last week | 11 (73) |
| B, Several times during the last week | 2 (13) |
| C, Every day | 2 (13) |
| D, Every day and night | 0 |
| E, Constant pain during the last week (day and night) | 0 |
| **7. How long does the pain persist when experienced this last week?^2^** |  |
| A, A few minutes | 14 (78) |
| B, Several minutes | 2 (11) |
| C, Most of the day | 1 (6) |
| D, All day | 1 (6) |
| E, Constant pain during the last week (day and night) | 0 |
| **8. Do you find it difficult to rise from a low-sitting chair as a result of your abdominal pain?^2^** |  |
| No | 17 (94) |
| Yes | 1 (6) |
| Not sure | 0 |
| Never perform this activity | 0 |
| **9. Do you find it difficult to sit for an extended period (over 30 minutes) as a result of your abdominal pain?^2^** |  |
| No | 16 (89) |
| Yes | 1 (6) |
| Not sure | 1 (6) |
| Never perform this activity | 0 |
| **10. Do you find it difficult to stand for an extended period (over 30 minutes) as a result of your abdominal pain?^2^** |  |
| No | 14 (78) |
| Yes | 4 (22) |
| Not sure | 0 |
| Never perform this activity | 0 |
| **11. Do you find it difficult to climb stairs as a result of your abdominal pain?^2^** |  |
| No | 16 (89) |
| Yes | 2 (11) |
| Not sure | 0 |
| Never perform this activity | 0 |
| **12. Do you find it difficult to drive a car as a result of your abdominal pain?^2^** |  |
| No | 17 (94) |
| Yes | 0 |
| Not sure | 0 |
| Never perform this activity | 1 (6) |
| **13. Has abdominal pain limited your ability to perform sports activities?^2^** |  |
| No | 14 (78) |
| Yes | 4 (22) |
| Not sure | 0 |
| Never perform this activity | 0 |
| **14. Have you taken any pain medication during the last week for abdominal pain?^2^** |  |
| No | 15 (83) |
| Yes | 3 (17) |
| **15. To what extent has abdominal pain limited your ability to work during the last two months?^2^** |  |
| A, I have not needed any sick leave as a result of abdominal pain | 17 (94) |
| B, Abdominal pain has caused 1-7 days of sick leave during the last 2 months | 0 |
| C, Abdominal pain has caused 1-4 weeks of sick leave during the last 2 months | 0 |
| D, Abdominal pain has caused constant sick leave during the last 2 months | 0 |
| E, Abdominal pain has caused me to seek disability income | 0 |
| F, I am unemployed or retired | 1 (6) |
| **16. Have you had hernia or any other type of abdominal surgery after your initial surgery?**  Yes | 6 (15) |
| **17. Do you feel any abdominal stiffness or rigidity after surgery?**  No  Yes | 18 (46)  21 (54) |
| **18. Are you satisfied with your operation?**  No  Yes | 5 (13)  34 (87) |
| **19. Would you repeat the operation if necessary?**  No  Yes | 2 (5)  37 (95) |
| **20. How would you describe your work?** |  |
| A, Heavy physical work | 5 (13) |
| B, Light physical work | 14 (36) |
| C, Office work | 20 (51) |

^1^ Data available for 15 patients. ^2^ Data available for 18 patients.
